# Analysis of Amyloid-β Pathology Spread in Mouse Models Suggests Spread Is Driven by Spatial Proximity, Not Connectivity

**DOI:** 10.3389/fneur.2017.00653

**Published:** 2017-12-18

**Authors:** Chris Mezias, Ashish Raj

**Affiliations:** ^1^Department of Neuroscience, Weill Cornell Medicine of Cornell University, New York, NY, United States; ^2^Department of Radiology, Weill Cornell Medicine of Cornell University, New York, NY, United States

**Keywords:** connectomics, neurodegenerative diseases, neurodegeneration, computational modeling, proteinopathy, amyloid, amyloid spread

## Abstract

While the spread of some neurodegenerative disease-associated proteinopathies, such as tau and α-synuclein, is well studied and clearly implicates transsynaptic pathology transmission, research into the progressive spread of amyloid-β pathology has been less clear. In fact, prior analyses of transregional amyloid-β pathology spread have implicated both transsynaptic and other intracellular- as well as extracellular-based transmission mechanisms. We therefore conducted the current meta-analytic analysis to help assess whether spatiotemporal amyloid-β pathology development patterns in mouse models, where regional proteinopathy is more directly characterizable than in patients, better fit with transsynaptic- or extracellular-based theories of pathology spread. We find that, consistently across the datasets used in this study, spatiotemporal amyloid-β pathology patterns are more consistent with extracellular-based explanations of pathology spread. Furthermore, we find that regional levels of amyloid precursor protein in a mouse model are also better correlated with expected pathology patterns based on extracellular, rather than intracellular or transsynaptic spread.

## Introduction

Spreading protein pathology is hypothesized by many scientists to underlie the spatiotemporal pattern of lesions (1), regional neuronal and volume loss (2, 3), as well as the progression of the presentation of symptoms (4) in degenerative diseases. Amyloid-β, the misfolded protein cleaved from amyloid precursor protein (APP), is the key constituent of amyloid plaques seen in Alzheimer’s disease (AD) and is thought to contribute to the observed toxicity and cell death along with tau (5). More current research has shown both misfolded tau and α-synuclein exhibit progressive protein pathology with prominent lesions progressing in fairly consistent spatiotemporal patterns (6, 7). However, the spread of amyloid-β pathology has not been neatly characterized into stages of pathology, with little evidence of focal seeding as is seen for tau or α-synuclein. However, studies tracking the spatiotemporal development of amyloid pathology have noted early development generally throughout the neocortex (8) with the deposition perhaps initially focused in posterior cortical areas (9). The staging research for amyloid pathology development generally shows more variance than do tau or α-synuclein staging for particular diseases and generally develops in a less focal manner.

The more focal and ordered staging characterizations for tau and α-synuclein, and perhaps underlying biological progression of pathology, have led to more successful study of the potential mechanisms of pathology spread for these proteins. For example, a whole litany of patient (3, 10) and mouse model-based (11–15) studies has looked at whole brain and cellular level resolutions and determined that the evidence, regardless of resolution of study, is most consistent with a transsynaptic and intracellular mechanism of tau spread. In fact, our own recent research demonstrates that tau pathology in mouse models progression mirrors the network of mouse fiber tracts (16). Similarly, regarding α-synuclein, there is strong evidence pointing toward transsynaptic and intracellular mechanisms of spread, including transfer of pathology from Parkinson’s and LBD patient neurons into grafted and implanted cells (17), the presence of both misfolded and normal state α-synuclein at the synapse (18), and controlled cell-to-cell transfer experiments in mice (18) demonstrating that α-synuclein spread occurs transsynaptically by virtue of initiation into a set of interconnected cells. The success in characterizing the spread mechanisms and abilities of tau and α-synuclein are now leading to efforts to slow down the progression of proteinopathy as a potential avenue for treatment with conditions based on both of these proteins (19, 20). However, the study of mechanisms of cell-to-cell or region-to-region transfer for amyloid-β pathology has produced mixed results.

The spread mechanisms of amyloid-β pathology, in terms of causing a misfold in healthy amyloid-β cleavage product, are well characterized and relatively similar to those for tau. The most accepted model, supported by evidence from patients (21), mouse model, and cell lines (5, 22), is that misfolded amyloid-β can recruit and induce misfolding in healthy protein product amyloid-β. However, unlike the cases of tau and α-synuclein, this finding has not helped gain more clarity in how amyloid-β pathology spreads transcellulary and transregionally. Some studies have proposed extracellular mechanisms of spread (23, 24), while other research has implicated internalization of amyloid-β as a key element in this protein product becoming pathological (5), with other researchers building on this finding and demonstrating potential transsynaptic and intracellular spread (25, 26). Given the failure of clinical trials targeting already misfolded amyloid-β (27, 28), we undertake the present research to help resolve inconsistency in understanding the pathology spread mechanisms of amyloid-β, with an eye toward beginning to make targeting of amyloid-β pathology spread mechanisms an even more viable therapeutic option.

In the present study, we hope to add clarity to the amyloid-β pathology spread debate by analyzing available data on the patterns of regional amyloid pathology severity and spatiotemporal progression in mouse models. We first demonstrate amyloid-β pathology patterns in mouse models are not mirrored by the mouse connectome, while those of tau pathology, accordant with prior research (16), are. Furthermore, a model of connectivity-based amyloid-β pathology spread fails to recreate spatiotemporal patterns of amyloid-β pathology development in a mouse model. However, we demonstrate that a model of extracellular, spatial proximity-based diffusion (SPD), does recreate the regional severity and spatiotemporal progression patterns of amyloid-β pathology. Moreover, spatial proximity to amyloid-β pathology initiating regions serves as a good proxy for predicting the regional severity of said pathology. We conclude that the spread of amyloid-β pathology is predominantly driven by extracellular means and that the basis for regional vulnerability to amyloid-β is how spatially proximal that region is with those already exhibiting pathology.

## Materials and Methods

### Study Selection for Datasets

Studies were found from a literature search of Web of Science and Google Scholar and had to meet the following criteria to merit inclusion: they had to be from 2005 onward to assure their methodology, and measurements were somewhat current with the standards in the field, they had to include data about the regional distribution of amyloid pathology from at least 5 different regions, and they had to be from mouse models using the APPswe mutation and had to assess endogenous pathology development, rather than pathology development induced from exogenous amyloidogenic seeds. We note that this criterion excludes some important studies, such as Ye et al. (24), but uses it nonetheless for consistency in our analyses. Amyloidopathy data here come from Harris et al. (25) (seven total regions and three timepoints), Bero et al. (26) (five total regions, one timepoint), and Lim et al. (29) (five total regions and one timepoint). Tau data cited for comparison purposes in Figure 2 came from Ahmed et al. (12) (5 total regions, 1 timepoint), Bolmont et al. (30) (5 total regions, 1 timepoint), and Clavaguera et al. (11) (11 total regions, 3 timepoints, only the final one used); please refer to the original citations for specific methodological questions about any of the above studies.

### The Allen Brain Institute Mouse Connectivity Atlas and Generating the Directed, Undirected, and Spatial Distance Graphs of the Mouse Brain

**Figure 1:**
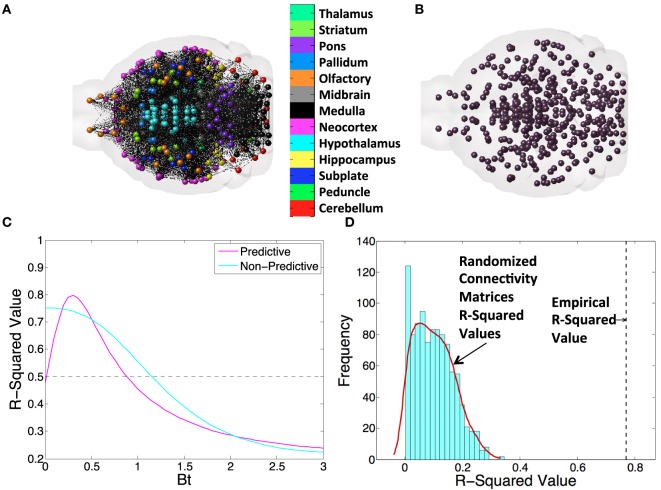
An example figure demonstrating all of the metrics and networks we use throughout the present study. We first illustrate the concept of a **(A)** connectivity network and **(B)** the centers of mass of gray matter regions and the distances between them to illustrate the basis of all connectivity and spatial diffusion based spread models. **(C)** Here, we exhibit a βt-curve, where βt is the time diffusion constant. Note that we find the value of best fit, as indicated, and subtract, as indicated, to get a Δ*r*-squared value. We use this value to compare across all DNT, NT, and spatial proximity-based diffusion (SPD) models in this study. **(D)** We also randomize matrices 1,000× when we use DNT or SPD to provide another metric of how unexpected our observed *r*-squared values would be given chance alone.

Connectivity and spatial distance data were taken from the supplementary dataset published along with the mesoscale mouse connectome from the ABI (31). Total projection volume between regions was generated by multiplying element-wise by the rows the connectivity matrix times the number of voxels in each seeding region. Regional distances used were the Cartesian distances between the centers of mass of any region pairs. For an anatomical illustration of the brain represented as a connectivity graph, see Figure 1A; directional connectivity from to and from the entorhinal cortex (EC), used as an example region, can be seen in Figure 1B. For an example of a spatial distance graph as a matrix, see Figure 1C.

### Network Laplacian and Its Eigenvectors

For a given connectivity *C* or distance matrix *S*, we define a Normalized Laplacian Matrix, as in Raj et al. (2), *L*:

$$L=I-D_{\text{r}}\cdot C\cdot D_{\text{c}}$$

where *D*_r_ and *D*_c_ are diagonal degree matrices of the sum totals of the rows and columns, respectively, and *I* signifies the identity matrix. In our notation for the connectivity matrices, *C* represents the retrograde transmission mode, and the corresponding anterograde mode is then simply given by *C*^T^, and the bidirectional mode by (*C* + *C*^T^)/2. The distance matrix was obtained from a separate dataset appended to Ref. (31) and is referred to in this manuscript using *S*. Normalized Laplacian matrices were generated for each mode with the above equation.

### Creating a Dynamic Model of Amyloid Pathology Spread Over Time

A previous graph theoretic model of pathology spread in AD throughout a brain network was shown to be predictive of future patterns of disease progression (2). The principle is to seed a graph node corresponding to a brain region with an arbitrary value and then model the diffusion of the disease factor throughout the network *via* the Network Diffusion equation:

(1) X(t)=exp(−βLt)⋅X(0).

This models the long-range patterns of spread of the protein pathology at any time *t* as a product of the initial seeding pattern *X*(0), and the so-called diffusion kernel exp (−β*tL*), with diffusion constant, β (2).

The major differences with previous network diffusion model are twofold: (1) we are for the first time using this model with a directed brain network and (2) we are interested in total pathology accumulation over time, which we model as a summative process:

(2) X(i)=exp(−βLΔt)⋅X(i−1)+X(i−1).

We use Eq. 2 to calculate, for any point in time, the deposition of tau, amyloid, and neuronal atrophy across the brain regions represented in the network. The Eq. 1 in the present study will be referred to as the standard undirected network diffusion model, or NT, while our modified directional version in Eq. 2 will be referred to as directed network transmission, or DNT. We discuss this equation modeling spatial pathology diffusion in the Section “Spatial Diffusion Modeling As an Alternative Model to Network Diffusion.”

### Spatial Diffusion Modeling As an Alternative Model to Network Diffusion

We additionally created a spatial diffusion model as a comparison or alternative hypothesis to the network diffusion model. The spatial diffusion model was based on the same fundamental network diffusion Eq. 2 explicitly stated above. The difference between DNT (and NT) and spatial diffusion in the present study is that the network for spatial diffusion is a matrix where each entry in the matrix *S_i,j_* is the reciprocal of the Cartesian distance between the center of mass of each GM region included in the Allen Institute’s mouse connectivity atlas. Using this distance matrix, *S*, rather than the connectivity matrix *C*, we ran the diffusion equation stated above in (2) to get a model approximating diffusion based on spatial proximity, which will be referred to as SPD.

### Comparing the DNT/NT and SPD Models with Previously Published Results and Examining the Question of Seeding

We ran the network and spatial diffusion Eq. 2, through the number of iterations, in months, given in each study. If a study measured pathology at 6 months, we ran the model through 6 iterations, and if a study measured pathology at 9 months, we ran the model through 9 iterations. The implicit assumption here is that amyloidopathy spreads in all datasets at the same rate. While these datasets were obtained with different mouse models potentially exhibiting different amyloid strains, we do not have enough data to assess the kinetics or speed of amyloid pathology spread in individual cases. Hence, we decided to impose a minimal and general set of assumptions across the board. In these iterations, we used Δ*t* = 1 month and modified β to achieve the optimal match with the data, as the empirical diffusion constant for various pathologies in AD is *a priori* unknown. Example, βt curves for a range of β values, where the model both shows behavior that is predictive of proteinopathy spread and non-predictive of proteinopathy spread can be seen in Figure 1C. We show anatomical examples of a graph that is the basis of NT/DNT in Figure 1A, and a model of the brain demonstrating the locations of regional centers of mass, which form the basis of the network used in SPD, in Figure 1B. To compare whether DNT/NT or SPD performed better at recapitulating amyloid-β pathology, we calculate Δ*r*-squared values, which are the difference between the *r*-squared value between the amyloidopathic seed (no diffusion) and empirical amyloidopathy, and the same *r*-squared value calculated between the best fit value of the βt model constants of diffusion time and empirical amyloid-β pathology. An example of this calculation can be seen in Figure 1C. Given the relatively small sample size of regions quantified from Harris et al. (25), we performed an additional assessment for the significance of regressions that were significant at an alpha of *p* < 0.05: we randomized either the connectivity or interregional distance matrices from the ABA 1,000 times and ran our models to produce simulated data generated with randomized networks, generating a distribution *r*^2^-values to use as a comparison with *r*^2^-values from our models run using empirical networks or distances. An example of the results of this process can be found in Figure 1D.

## Results

The current paper attempts to perform a whole-brain scale, meta-analytic study of amyloid-β pathology spread mechanisms. The motivation behind such research is clear: while research into other proteinopathies with prion like qualities, such as tau pathology, has demonstrated a clear mechanism and clear pattern of transmission, such as transsynaptic spread (1), efforts to characterize misfolded Aβ spread are conflicted (24–26). Indeed, understanding misfolded amyloid spread mechanisms as Aβ proteinopathy develops could prove a fruitful avenue of research for future disease course modifying treatments if further amyloid transmission can be prevented. We first demonstrate that given three studies regionally quantifying tau pathology, we see clear evidence of transsynaptic transregional spread, but that such evidence is lacking with respect to amyloid pathology transmission in another three studies. We then demonstrate, using models of both progressive spread over the brain’s connectivity network (NT and DNT) and *via* diffusion based on spatial proximity to already affected regions (SPD), that diffusion to spatially proximate regions is a much better characterization of both the development of Aβ pathology and of the regional pattern of Aβ precursor protein, APP, levels. The present results are discussed in detail in the subsections below.

### Amyloid-β Pathology, Unlike Tau Pathology, Is Not Well Characterized in Mouse Models by the Brain’s Connectivity Network

**Figure 2:**
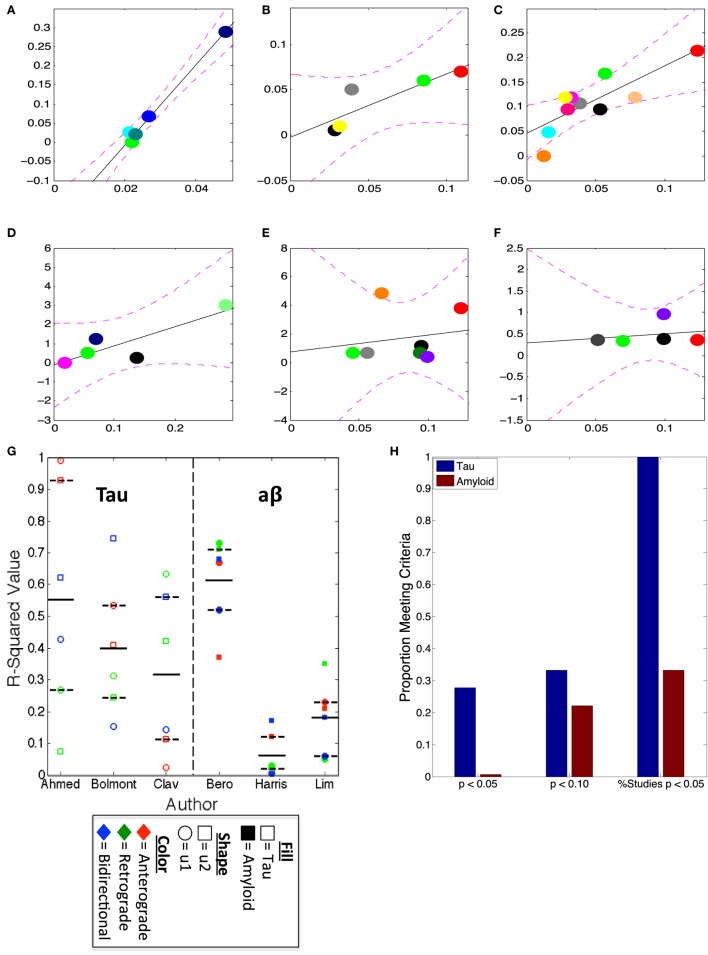
*u1* and *u2* consistently predict regional tauopathy, but not amyloidopathy, severity. **(A–F)** The best regressions, by *r*^2^-values, using the anterograde, retrograde, and bidirectional Allen Institute mouse brain connectivity networks, plotted, of *u1* or *u2* vs. deposition data from several studies; **(A)** is the regression between anterograde *u1* and Ahmed et al. (12), **(B)** is between bidirectional *u2* and Bolmont et al. (30), **(C)** is retrograde *u1* vs. Clavaguera et al. (11), **(D)** is the regression of retrograde *u1* and Bero et al. (26), **(E)** is between bidirectional *u2* and Harris et al. (25), while **(F)** is Lim et al. (29) vs. retrograde *u2*. All *r*^2^-values for all *u1* and *u2* regressions vs. each dataset can be found in Table 1. **(G)** The scatter plot of *u1* and *u2*, for each direction, *r*^2^-values with actual data indicates that at least one direction’s eigenvectors, if not multiple, show high correspondence with tau deposition, but that only the data from Bero et al. (26), is correlated with the eigenvectors from the directed connectivity graphs. **(H)** This bar graph illustrates the consistent pattern of tau and eigenvector correspondence, and the relative inconsistency of correlations between amyloid and eigenvectors. The first *x*-label in **(H)** refers to the proportion of eigenvectors, regardless of study and first of second eigenvector, yielding a *p* < 0.05, the second those yielding *p* < 0.10, and the third refers to the % of studies used here having any eigenvector yielding significant results at the threshold *p* < 0.05.

Prior use of the brain connectome graph metrics and models (such as DNT and NT) have successfully, characterized the spatiotemporal development of regional volume loss (2) and metabolic deficits (3) in patients, and the spatiotemporal proliferation of tau pathology in mouse models (16). We use the first two eigenvectors of the mouse brain connectome to assess whether amyloid-β pathology can be recapitulated by the properties of mouse brain wiring in a way that resembles the accurate characterization of tau pathology. We find that, while, accordant with prior research (16) relative regional severities of tau pathology across all three studies highlighted here [Figures 2A–C,G,H; Table 1; (11, 12, 30)] are all strongly recapitulated by at least one the first two eigenvectors of all of the mouse connectomes used (anterograde, retrograde, and bidirectional), only one study regionally quantifying amyloid pathology produced any such significant results [Figures 2D,G,H; Table 1; (26)]. The results comparing the eigenvectors from our mouse connectomes with our other two amyloid datasets (25, 29) showed no significant match between where, based on transsynaptic spread, one would expect the most severe amyloid pathology and where the empirical dataset indicated the most severe pathology, across all mouse connectomes (Figures 2E–H; Table 1). The present results confirm our prior analyses that tau pathology patterns in mouse models can be well characterized using mouse connectomes but also reveal that no such strong relationship exists between amyloid-β pathology patterns in mouse models and the mouse connectomes.

**Table 1:** Connectivity network *u1* and *u2* consistently mirror regional tau, but not Aβ, pathology patterns.

CON. *u1* vs. pathology	Pathology measure	Eigenvector	Anterograde	Retrograde	Bidirectional

Tau pathology					
Ahmed et al. (12)	% AT8 (+) cells	*u1*	0.98**	0.28	0.43
		*u2*	0.92**	0.08	0.61
Bolmont et al. (30)	% AT8 (+) cells	*u1*	0.53^	0.32	0.16
		*u2*	0.41	0.25	0.75*
Clavaguera et al. (11)	Tangle BURDEN	*u1*	0.04	0.63**	0.14
		*u2*	0.12	0.43	0.56*

**Aβ pathology**					

Bero et al. (26)	% Area plaques	*u1*	0.66^	0.73^	0.52^
		*u2*	0.37	0.72^	0.68^
Lim et al. (29)	% Area plaques	*u1*	0.04	0.06	0.01
		*u2*	0.12	0.05	0.17
Harris et al. (25)	Plaque burden	*u1*	0.24	0.07	0.07
		*u2*	0.21	0.35	0.18

In the table above, all *r*-squared values between both of the first two eigenvectors of each connectivity network, anterograde, retrograde, and bidirectional, and empirical tau and Aβ pathology patterns are reported, with the study indicated in the leftmost column. ^*p* < 0.10, *p < 0.05, **p < 0.01.

### Spatiotemporal Amyloid-β Pathology Is Better Characterized by a Model of Spatial, Rather Than Connectome Based, Diffusion

**Figure 3:**
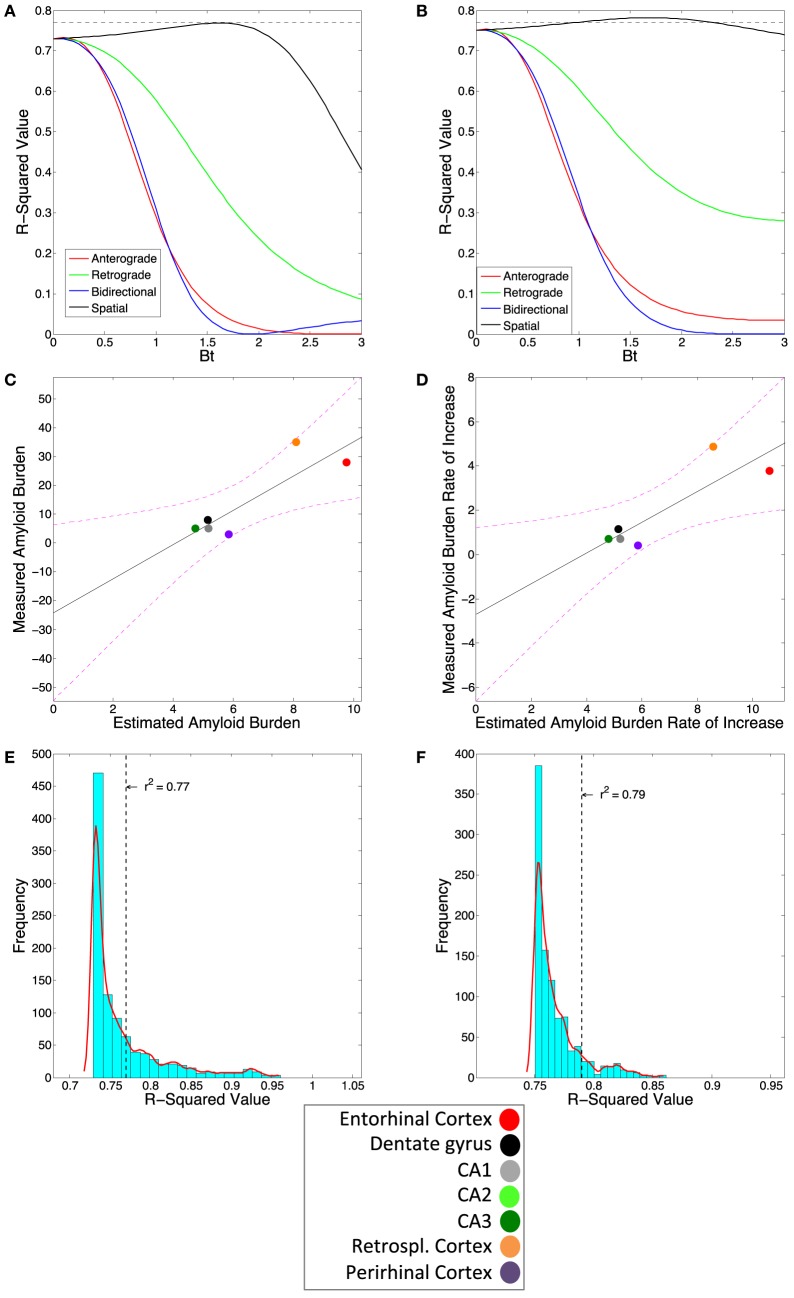
Amyloidopathy is not predicted by DNT or NT but rather by spatial proximity-based diffusion (SPD). **(A)** The βt vs. *r*^2^-value curve comparing the three DNT/NT and the SPD models’ predictions to regional rate of increase and **(B)** end-state regional amyloid plaque burden data from Harris et al. (25). Note all three DNT/NT models’ curves are non-predictive. **(C)** The plotted regression of the SPD model against the regional rate of increase and **(D)** end-state regional amyloid burden data. The *r*^2^-value from the SPD model using the Cartesian distance between region matrix performs better than most, but not all, randomized distance matrices for both **(E)** regional rate of pathology increase and **(F)** end-state regional amyloid burden data. Color legends are included. All exact *r*-squared values can be found in Table 2.

We next assess how well our connectivity based DNT/NT model recapitulates the longitudinal spatiotemporal development of amyloid-β pathology in our one multi-timepoint mouse amyloid dataset from Harris et al. (25). We find that, at no measured timepoint either early or late, does anterograde or retrograde DNT or bidirectional NT successfully predict amyloid pathology patterns (Table 2). In fact, at all timepoints, including the final measured timepoint, DNT and NT fail to add any information beyond the EC seeding reported by Harris et al. (25) for predicting the development of amyloid-β pathology (Figure 3A). The regional slopes of pathology severity increase show an identical pattern of results (Figure 3B).

**Table 2:** DNT and NT do not predict either Aβ pathology or amyloid precursor protein (APP) levels, at any timepoint measured in Ref. (25), but spatial proximity-based diffusion (SPD) does across all timepoints.

NT and SPD vs. Aβ path	Model assessor	Ant. DNT	Ret. DNT	Bidi. NT	SPD
Harris et al. (25) Aβ T1	Δ*R*	0.00	0.00	0.00	0.06
Harris et al. (25) Aβ T2	Δ*R*	0.00	0.00	0.00	0.05
Harris et al. (25) Aβ slope	Δ*R*	0.00	0.00	0.00	0.04
Harris et al. (25) APP	Δ*R*	0.00	0.02	0.00	0.04

*This table reports ΔR-squared values, which is the change in r-squared between baseline and the best-fit each model produce produced. Only SPD produces values > 0 across all measurements*.

However, the amyloid-β pathology patterns at all timepoints, including the final measured timepoint, are significantly recreated using the SPD model of pathology spread, which is based on spatial proximity to the EC, where pathology initiated (Figures 3A,C; Table 2). Moreover, the regional slopes of amyloid-β pathology severity increase are also significantly predicted by SPD (Figures 3B,D; Table 2). Due to the relatively small sample size of mice and quantified regions from Harris et al. (25), we tested whether the results from the SPD model were due to chance by assessing whether significant results could be obtained using randomized distances between regions; we found that for the final measured timepoint of amyloid-β pathology, SPD using empirical distances between brain regions outperformed 91% of the 1,000 randomizations, and 93% of the randomization when predicting the regional severities of rate of amyloid-β pathology increase (Figures 3E,F).

### Regional Levels of APP Are Better Predicted *via* a Model of Spatial, Rather Than Connectome Based, Diffusion

**Figure 4:**
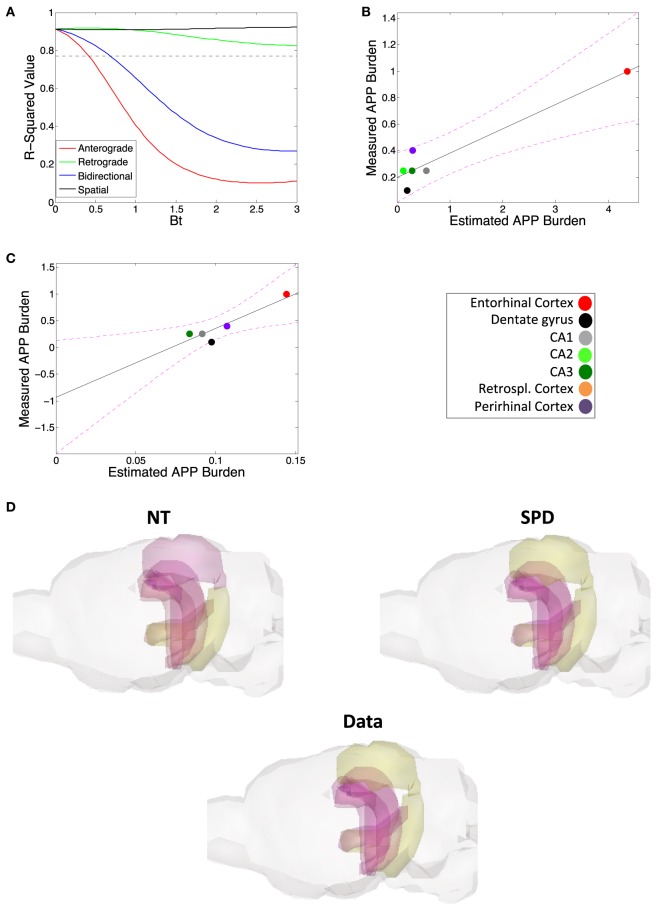
Amyloid precursor protein (APP) regional deposition patterns are, akin to amyloid burden, well modeled by spatial proximity-based diffusion (SPD). **(A)** The βt vs. *r*^2^-value curve comparing the predictions of the DNT/NT and SPD models regional APP deposition data from Harris et al. (25). **(B)** The plotted regression of retrograde DNT’s predictions vs. empirical APP regional deposition data. Note that the regression may largely be driven by the entorhinal cortex as an outlier, calling into question whether DNT diffusion is predictive. **(C)** The plotted regression of the SPD model’s predictions vs. empirical APP regional deposition data. Note that this regression is both significant and not driven by an outlier. **(D)** We also include an anatomical illustration of empirical pathology (labeled “Data”) as well as the predictions of the NT and SPD models, of regional rates amyloid pathology increase from the initial regional APP measurement to the final measured timepoint of amyloid pathology, from Harris et al. (25). In this illustration, yellow is more severe and purple more mild pathology. Color legends are included. All *r*-squared values are in Table 2.

The data from Harris et al. (25) also includes regional quantification of amyloid-β precursor protein (APP) levels using anti-hAPP antibody. While we find that amyloid-β pathology proliferation is not well explained by connectome-based spread models, but rather by spatial proximity-based models, we want to test whether regional levels of APP can be predicted using the connectome. However, our results again indicate that, while retrograde DNT does give a significant prediction of APP levels (Figures 4A,B), anterograde DNT and bidirectional NT do not, and SPD outperforms even retrograde DNT at recreating relative regional levels of APP (Figures 4A,C; Table 2). Furthermore, the significant match between the predictions of retrograde DNT and regional APP levels appears to be driven by a single outlier region (Figure 4B), calling the validity of these results into question. Akin to our results about the spatiotemporal development of amyloid-β pathology in the subsection above, we find that regional levels of APP are again best predicted by a model of diffusion based on spatial proximity with regions exhibiting high APP levels, more accordant with extracellular diffusion mechanisms than connectivity based or transsynaptic based proliferation. To emphasize the above results, we also include an anatomical illustration of the regional rates of suspected pathology increase, from the measurement of regional APP levels until the final quantification of amyloid-β pathology can be seen in Figure 4D; note that SPD is a better model for spatiotemporal amyloid-β pathology patterns than is DNT/NT.

## Discussion

While research into other protein pathology spread mechanisms in mouse models, such as work regarding synucleinopathy (18) and tauopathy (11–16) reveal clear pathology development patterns mirroring anatomical connectivity networks and strongly suggest transsynaptic spread, no such clarity exits regarding spread patterns and mechanisms for misfolded amyloid-β. What is clear however is that amyloid pathology does spread outward in a manner that is highly dependent on the brain region into which amyloidogenic seeds are placed, even when using the same amyloid pathology source and transgenic mouse model (32, 33).

We accordingly undertook the present analysis to determine whether we could find clear evidence of transsynaptic spread or pathology development mirroring connectivity networks for amyloid-β, as some prior research has claimed (25, 26), or whether some other pattern and mechanism of spread better explains amyloid-β pathology development (24, 29). We first demonstrate that, especially compared with tau pathology development, amyloid-β pathology, across studies, is not most pronounced in regions that are most likely to accumulate pathology if spread is *via* the mouse anatomical connectivity network (Figure 2; Table 1). We next demonstrate that, in assessing our lone longitudinal dataset (25) a model based on spread to spatially proximal (SPD), rather than a model assuming transmission to interconnected (NT/DNT) regions, better recreates the spatiotemporal patterns of amyloid-β pathology development and the relative regional severities of amyloid-β plaques (Figures 3 and 4; Table 2). These results and their further implications are discussed in detail in the subsections below.

### Pathology Spread into Spatially Proximal, but Not Necessarily Highly Interconnected Regions, Implies Extracellular Spread

The present analysis shows results suggestive of amyloid-β pathology spread being predominantly extracellular, rather than intracellular. Modeling assuming transsynaptic spread failed to significantly recapitulate empirical patterns of amyloid-β pathology (Figures 2–4; Tables 1 and 2), while modeling based on spatial proximity significantly recreated observed spatiotemporal amyloid-β pathology development patterns (Figures 3 and 4; Table 2). While prior studies implicate transsynaptic amyloid-β pathology transmission (25, 26), these findings are contradicted by other research demonstrating a lack of evidence for transsynaptic or even any form of intracellular transneuronal and transregional spread (24, 29). By contrast, studies of other pathological protein species’ mechanisms of spread, such as pathological tau, consistently implicate transsynaptic spread (11–16) with little evidence for other mechanisms.

Extracellular diffusion resulting in amyloid-β pathology transmission to the areas of the brain most spatially proximal to those already exhibiting amyloidopathy fits with the known characteristics of amyloid-β as a protein. First, APP, the precursor protein for amyloid-β, is a transmembrane protein with a large extracellular domain, and it is from this large extracellular domain that amyloid-β is formed as a cleavage product from APP (34). Post-cleavage, amyloid-β generally exists in the extracellular space, even as a healthy protein (35). Finally, amyloid-β in its pathological states also forms plaques almost exclusively extracellularly (36). None of this is to say that pathological amyloid-β might not gain capabilities of becoming internalized into neuron or spreading transneuronally or transsynaptically, as some authors have suggested (25). But given our meta-analytic results suggesting no mathematical basis for transsynaptic spread or any spread based on the brain’s connectivity network, and suggesting that pathology is more likely to spread into regions spatially close to rather than heavily interconnected with already affected areas, we posit that our present work, strongly implies some mechanism of pathology spread that is extracellular. We hope that our work serves as a starting point for more heavily quantitative investigations of amyloid-β pathology transmission with an aim toward clearing up the inconsistent results about how and where amyloid-β progresses around the brain.

Given that amyloid patterns are known to depend on exogenous seeding site (32, 33), a purely spatial mode of spread would suggest a radial distribution of pathology spread, which was not observed in our data. However, there is very little evidence that initial amyloid seeding is in fact focal; instead early amyloid pathology is found diffusely in the neocortex and then spreads to subcortical areas (8). Quite likely, amyloid pathology could be caused or initiated by metabolic deficits that target particular regions, leading to amyloid initiation in broad areas of the brain, for instance in the default mod network (9). Due to broad initiation, a radial spread from a focal source may not be manifested under spatial spread. Nonetheless, it is unlikely that spatial spread *via* extracellular spaces is the only process involved in amyloid pathology ramification. Several additional factors might play a role beyond pure spatial spread, and future research will be needed to identify and assess them.

Also of note is that our SPD model accurately recreated regional APP levels from Harris et al. (25). Given that APP is a transmembrane protein (34), spread based on spatial proximity, such as through the extracellular space, seems unlikely. First, it is possible that the anti-hAPP antibody used in the study (25) bound to a non-Aβ fragment of APP following cleavage, sAPPα, which akin to Aβ is also often released into the extracellular space (34) and could therefore plausibly spread into spatially proximal regions. Aβ also has known reactions with full-length transmembrane APP (34), and so spatially spreading pathological Aβ could influence regional APP levels by upregulating APP expression or by inducing the release of transmembrane APP into the extracellular space. These hypotheses for why a model of diffusion into spatially proximal regions accurately captured empirical APP levels, however, are conjecture at this point. Akin to the discussion of factors beyond spatial proximity contributing to the spatiotemporal development of Aβ pathology, above, elucidating why APP levels mirror Aβ pathology spread patterns in at least a pathological mouse model, will require careful future research.

### Extracellular Spread of Amyloid-β Pathology Fits with Known Synaptic Problems Resulting from Its Pathology

Deficits in cellular function directly attributable to amyloid-β in mouse models are generally synaptic deficits (37, 38). Given the propensity for amyloid-β plaques to accrue at synapses (39), it is not surprising that such an accumulation of misfolded protein species would causes deficits in the ability to transmit and receive action potentials (37, 38). However, our findings implying extracellular spread based on spatial proximity to already affected regions, coupled with the above work demonstrating the propensity for amyloid-β pathology to cause synaptic and cell-signaling deficits raise an important question: why should misfolded amyloid-β, if it is spreading and circulating extracellularly, preferentially cause issues at synapses, rather than at other areas along the very large neuronal membranes?

Work whose data are used in the present analysis regarding spatiotemporal amyloid-β pathology development provides a possible explanation. Some recent studies demonstrate amyloid-β pathology appears more likely to accrue in more active regions (26) close to those already exhibiting pathology. Furthermore, this work suggests electrical signaling between neurons may act as an attractant for pathological amyloid-β (26), inducing more amyloid-β to accumulate and therefore form plaques at cellular locations where electrical current leakage is the greatest: at the synapse (39). If, as our results indicate, amyloid-β pathology follows a pattern of transiting to spatially proximal, rather than interconnected areas, likely *via* some form of extracellular diffusion, then electrical current leakage from the synapse could bias amyloid-β pathology spread toward those regions that are both spatially proximal and more likely to have high signaling (and therefore electrical) activity. This could further explain why, even though SPD consistently outperformed DNT/NT, it was still not as strong a model for predicting spatiotemporal amyloid-β pathology development as DNT/NT are for modeling tau pathology (16). Amyloid-β pathology spread patterns might be best explained by a model incorporating both spatial proximity with already affected regions and the average signaling activity of those spatially proximal regions; our research suggests this as a topic for further study in elucidating amyloid-β transmission.

### Extracellular Spread of Amyloid-β Pathology Is Complicated by Hypotheses of Interactions with Known Intracellular Pathological Proteins, Tau, and α-Synuclein

While amyloid-β is mainly an extracellular protein, other protein pathologies known to be comorbid with amyloid-β pathology are caused by intracellular proteins tau and synuclein. A potential complication for the model of extracellular spread of amyloid-β pathology into the most spatially proximal regions is complicated by evidence demonstrating potential interactions between amyloid-β and tau (30, 40, 41) and known co-occurrence of misfolded amyloid-β plaques embedded within Lewy Bodies composed primarily of misfolded synuclein (42). Synuclein is almost exclusively present at synaptic terminals and tau is an almost exclusively axonal protein, so given the prior work cited above showcasing known amyloid-β propensity for accumulating around and causing issues at synapses (37–39), a predominantly extracellular mechanism of misfolded amyloid-β spread does not necessarily negate the possibility of the interaction of these proteins. However, our present work does not provide a satisfying explanation as to how pathological proteins that are on the one hand predominantly intracellular and on the other are the likely extracellular transiting amyloid-β are likely to interact. We nonetheless feel it is important to point out the limitation to our current work and to encourage future research to pursue this question.

## Author Contributions

No animal or human research subjects were directly used in the present research. All authors read and approved of the manuscript.

## Conflict of Interest Statement

The authors declare that the research was conducted in the absence of any commercial or financial relationships that could be construed as a potential conflict of interest. The reviewer RM and handling Editor declared their shared affiliation.
